# Impact of proton pump inhibitors on the onset of gastrointestinal immune‐related adverse events during immunotherapy

**DOI:** 10.1002/cam4.6565

**Published:** 2023-09-22

**Authors:** Angioletta Lasagna, Federica Mascaro, Simone Figini, Sara Basile, Giulia Gambini, Catherine Klersy, Marco Vincenzo Lenti, Antonio Di Sabatino, Alice Di Benedetto, Monica Calvi, Raffaele Bruno, Paolo Sacchi, Paolo Pedrazzoli

**Affiliations:** ^1^ Medical Oncology Unit, Fondazione IRCCS Policlinico San Matteo Pavia Italy; ^2^ Biostatistics and Clinical Trial Center, Fondazione IRCCS Policlinico San Matteo Pavia Italy; ^3^ Department of Internal Medicine and Medical Therapeutics University of Pavia Pavia Italy; ^4^ Department of Internal Medicine Fondazione IRCCS Policlinico San Matteo Pavia Italy; ^5^ Pharmacy Unit, Fondazione IRCCS Policlinico San Matteo Pavia Italy; ^6^ Division of Infectious Diseases I Fondazione IRCCS Policlinico San Matteo Pavia Italy; ^7^ Department of Clinical Surgical Diagnostic and Pediatric Sciences University of Pavia Pavia Italy

**Keywords:** colitis, hepatitis, immune checkpoint inhibitors, microbiome, proton pomp inhibitors, toxicity

## Abstract

**Introduction:**

The gut microbiota (GM) can influence the pathogenesis of immune‐mediated adverse events (irAEs). Proton pump inhibitors (PPIs) can affect the integrity of GM, but their role in promoting irAEs is still poorly understood.

**Methods:**

In this retrospective single‐center cohort study, the primary endpoint was the evaluation of the incidence of gastrointestinal (GI) irAEs in cancer patients on PPIs (exposed) versus cancer patients who were not on PPIs (unexposed).

**Results:**

Three hundred and sixty three patients' records (248 M/115F, median age 69) were reviewed. Twenty‐three exposed patients (92%) developed GI irAEs while only two unexposed patients (8%) developed GI irAEs (hazard ratio [HR] 13.22, 95% confidence interval [CI] 3.11–56.10, *p* < 0.000). This HR was confirmed after weighting for the propensity score (HR15.13 95% CI 3.22–71.03, *p* < 0.000).

**Conclusion:**

Chronic PPI use is associated with an increased risk of GI irAES.

## 1 INTRODUCTION

Immune checkpoint inhibitors (ICIs) play an increasingly relevant role in the management of solid tumors. Although the efficacy and durability of response with ICIs have been well established, one of the major issues is the high rate of immune‐related adverse events (irAEs) during the treatment.^1^ The mechanism of toxicity varies according to ICI, and may ultimately affect the overall management. Gastrointestinal (GI) toxicity is among the most frequently reported irAEs. Overt colitis occur in 8%–27% of the patients, while the incidence of diarrhea alone has been reported to be as high as 54% among the patients on ICIs.^2^ Hepatitis is described in 5%–10% of the patients during ICI monotherapy and in 25%–30% during a combination of anti‐CTLA‐4 and anti‐PD (L) 1.^3^

The intestinal toxicity of ICIs has been associated with gut microbiota (GM) alterations, particularly to a significant increase of *Bacteroides intestinalis*.^4^ More in depth, *Firmicutes* have been implicated in a higher incidence of irAEs while *Bacteroidetes* positively correlated with a lower incidence.^5^ The GM may therefore directly influence the pathogenesis of irAEs, and indirectly through the regulation of metabolites, cytokines, and immune cells.^6^ Moreover, the imbalance of gut‐liver axis caused by GM dysbiosis and/or gut mucosal barrier damage leads to various types of liver diseases,^7^ while a higher GM diversity seems to be a protective factor against irAEs.^6^ Indeed, the concomitant drugs given to these patients may alter GM diversity. For example, antibiotics can modify the GM composition, increasing inflammasome signaling, and thus promoting a pro‐inflammatory state, susceptible to GI irAEs.^8^

Proton pump inhibitors (PPIs) are among the most commonly prescribed drugs worldwide, although without evidence‐based indication in many cases.^9^ PPIs may favor the onset of immune‐mediated disorders via multiple mechanisms, including GM alterations, malabsorption of nutrients and vitamins, and possibly via other unknown effects.^10^ Indeed, PPI treatment was associated with distinct taxonomic alterations: in the upper GI tract, PPIs users showed an overgrowth of orally derived bacteria, mostly *Streptococcaceae*. In fecal samples, PPI increased multiple taxa from the orders *Bacillales* and *Lactobacillales*, the families *Pasteurellaceae* and *Enterobacteriaceae* and the genus *Veillonella*.^11^ Growing evidence shows that PPIs affect the integrity of GM and their use is consistently associated with profound changes in GM with a reduced α diversity.^12^

Despite the abovementioned premises, the role of PPIs in promoting irAEs has been poorly addressed. Hence, we sought to describe the incidence of GI irAEs in patients with solid tumors undergoing immunotherapy and concomitant PPIs.

## 2 MATERIALS AND METHODS

### 2.1 Study design and setting

In this retrospective single‐center cohort study, we reviewed all the patients with solid tumor who had received immunotherapy with or without chemotherapy at Medical Oncology Unit of Fondazione “IRCCS Policlinico San Matteo di Pavia” between January 2016 and October 2022. The study was conducted according to the Strengthening the Reporting of Observational Studies in Epidemiology (STROBE) Statement for reporting observational studies^13^ and was approved by the local Ethics Committee (Comitato Etico Area Pavia) and Institutional Review Board (P‐0004914/23) according to the ethical guidelines of the 1975 Declaration of Helsinki. All the subjects signed, before the initiation of treatment, an informed consent provided by the Fondazione IRCCS Policlinico San Matteo at hospitalization.

We defined “exposed” all the cancer patients who had received immunotherapy and concomitant PPIs, conversely all the cancer patients who had received immunotherapy without concomitant PPIs were defined ‘unexposed’.

The primary endpoint was the evaluation of the incidence of GI irAEs in the cancer patients who were on PPIs (exposed) versus the cancer patients who were not on PPIs (unexposed). The secondary objectives were: (i) evaluation of Overall survival (OS) between exposed and unexposed; (ii) evaluation of Progression Free Survival (PFS) between exposed and unexposed.

### 2.2 Data collection

Data were collected from the hospital's electronic patient records, including sociodemographic (age, sex) and clinical characteristics, such as type of cancer, TMN stage, treatment, the use of PPIs and/or antibiotics, the onset of IRAEs, the comorbidities (diabetes mellitus, heart disease, autoimmune diseases).

The inclusion criteria were: (i) patients aged 18 and older, regardless of gender; (ii) treatment with immunotherapy alone or in combination with chemotherapy; (iii) patients who received at least 3 months of ICIs; (iv) signing of informed consent. Patients with unavailable and incomplete basic characteristics, laboratory data, and follow‐up information under 3 months were excluded from the study.

### 2.3 Statistical analysis

All statistical analyses were performed with the Stata 17 (StataCorp.). A 2‐sided *p*‐value<0.05 was considered statistically significant. Continuous data were reported as median and quartiles (IQR); categorical data were reported as counts and percent. They were compared between cohorts with the Mann Whitney *U* test and the Fisher exact test respectively.

The incidence of GI irAEs was computed for each cohort as number of events per 100‐person year. The cumulative irAE‐free survival (defined as the time free from irAEs from the start of ICIs therapy) was computed and plotted using the Kaplan Meier method and compared using the logrank test. Hazard ratio (HR) and 95% Confidence Interval (CI) were derived from a Cox model. The proportional hazard assumption was satisfied. To adjust for the bias by indication a propensity score for using PPIs was estimated via logistic regression, including the following baseline patients' characteristics: age, BMI. History HIV, HBV or HCV infection, presence of comorbidities, type of tumor, metastases, tumor stage and oncologic treatment. The Hoteling test to compare distribution was nonsignificant (*p* = 0.559). The Cox model was then weighted using the inverse probability of PPI administration derived from the PS.

OS and PFS were analyzed as described for the primary endpoint.

The dataset generated from this study is not publicly available due to data protection compliance. The corresponding author upon reasonable request can share the raw data.

## 3 RESULTS

### 3.1 Characteristics of the study population

Three hundred sixty‐three patients' records (248 males [68.3%], 115 females, [31.7%]; M:F ratio 2.2:1; median age 69 years, IQR 61–76) were retrospectively reviewed. Median body mass index (BMI) was 24.22 (IQR 22.31–26.12). All the clinical characteristics that were retrieved are shown in Table 1.

**TABLE 1:** General clinical patients' characteristics.

Sex	Number of patients	%
Female/male	115/248	31.7/68.3
Comorbidities		
HCV	19	5.3
HBV	20	5.5
HIV	3	0.8
No comorbidities	141	38.8
≥1 comorbidity	220	60.9
Unknown comorbidities	2	0.5
Diabetes mellitus type 2	63	30.3
Cardiovascular disease	201	92.6
Autoimmune diseases	21	10.2

Abbreviations: HIV, human immunodeficiency virus; HBV, hepatitis B virus; HCV, hepatitis C virus.

One hundred seventy‐nine (49.4%) had non‐small cell lung cancer (NSCLC), 101 (27.9%) had melanoma, 32 (8.8%) had head & neck cancer, 29 (8%) had kidney cancer. The remaining 21 patients had bladder cancer (eight patients, 2.2%), squamous cell skin cancer (eight patients, 2.2%), small cell lung cancer (SCLC, three patients, breast cancer (one patient, 0.3%) and esophageal cancer (one patient, 0.3%). The most common treatment was ICI alone (321 patients, 88.7%) and the most common class of ICI was anti PD‐1 (324 patients, 89.5%; Table 2).

**TABLE 2:** Oncological patients' characteristics.

Type of tumor	Number of patients	%
NSCLC	179	49.4
Melanoma	101	27.9
Head & neck cancer	32	8.8
Kidney cancer	29	8.0
Bladder cancer	8	2.2
Squamous cell skin cancer	8	2.2
SCLC	3	0.8
Esophageal cancer	1	0.3
Breast cancer	1	0.3
Stage TNM		
Stage I	3	0.8
Stage II	0	0
Stage III	30	8.3
Stage IV	329	90.9
Type of oncological treatment		
ICIs	321	88.7
ICIs+Chemotherapy	36	9.9
ICIs + Targeted therapy	5	1.4
Type of ICIs		
Anti CTLA4	7	1.9
Anti PD‐1	324	89.5
Anti PD‐L1	23	3.6
Anti CTLA4 + anti‐PD‐1	18	5
Type of Anti‐PD‐1		
Nivolumab	163	50.3
Pembrolizumab	150	46.3
Cemiplimab	11	3.4
Type of Anti‐PD‐L1		
Atezolizumab	2	15.4
Avelumab	3	23.1
Durvalumab	8	61.5

Abbreviations: ICI, immune checkpoint inhibitors; NSCLC, non‐small cell lung cancer; SCLC, small cell lung cancer.

### 3.2 Exposure to PPIs and antibiotics therapy

**FIGURE 1:**
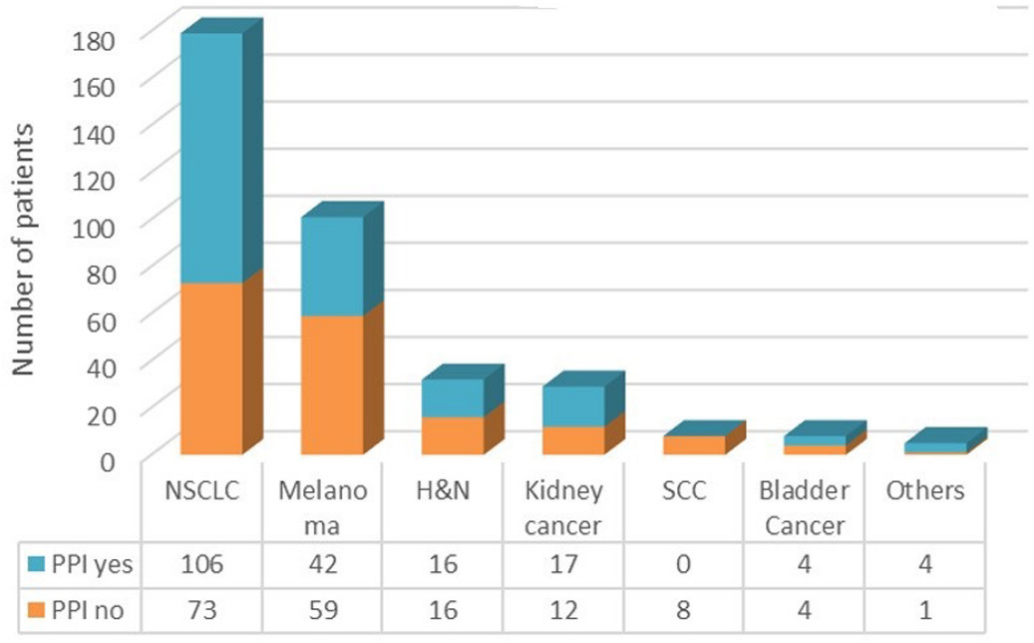
PPI exposed versus PPI unexposed according to the type of cancer.

One hundred and eighty‐nine (52.2%) patients were exposed to PPIs during the study period. The most common type of PPIs was pantoprazole (124 patients, 65.6%). A medical indication for the use of PPIs was not clearly reported in most of cases (130 patients, 68.8%), while the most common clinical indications were the age over 75 (29 patients, 58.5%), gastroesophageal reflux disease (GERD) (10 patients, 17%) and functional dyspepsia (10 patients, 17%). No patients at the time of the start of ICI were taking aspirin. The patients' characteristics according to PPI exposition are described in Table 3 and in Figure 1.

**TABLE 3:** Patients' characteristics PPI exposed versus PPI unexposed.

Variable	PPI unexposed (interquartile medians)	PPI exposed (interquartile medians)	*p* Value
Age	70 (60–78)	69 (61–67)	0.38
BMI	24.3 (22.4–25.76)	24.1 (21.9–26.5)	0.67
Male	123 (72.4%)	125 (66.1%)	0.12
Female	47 (27.6%)	64 (33.9%)	0.13
HIV	2 (1.2%)	1 (0.5%)	0.46
HBV	5 (2.9%)	15 (7.9%)	0.03
HCV	9 (5.2%)	10 (5.3%)	0.6
Comorbidities	93 (54.1%)	127 (67.2%)	**0.007**
Presence of metastases	153 (90.5%)	181 (95.8%)	0.04
Oncological therapy			0.3
ICIs alone	158 (91.3%)	163 (86.2%)	
ICIs + CTH	13 (7.5%)	23 (12.2%)	
ICIs + targeted therapy	2 (1.2%)	3 (1.6%)	
ABT	2 (1.2%)	6 (3.2%)	0.2

Abbreviations: ABT, antibiotic therapy; BMI, body mass index; CTH, chemotherapy; HIV, human immunodeficiency virus; HBV, hepatitis B virus; HCV, hepatitis C virus; ICI, immune checkpoint inhibitors.

Eight patients (0.6%) received antibiotic therapy within 30 days prior to ICI initiation. Six of these patients (75%) received both PPI and antibiotics.

### 3.3 Primary endpoint: irAES and exposure to PPIs

During the follow up period, 25 GI irAES were reported, in particular: diarrhea (*n* = 7, 28.0%), colitis (*n* = 12, 48.0%), gastritis (*n* = 1, 4.0%), hepatitis (*n* = 6, 24.0%). irAEs arised after a median of seven cycles of ICIs (IQR 4–12). A full list of toxicities and frequencies can be found in Table 4. For this work, we decided to focus only on GI irAEs, so the statistical analyses considered only such events.

**TABLE 4:** Toxicity events, by type and grade.

Toxicity events, by type	Any Grade	Grade 1	Grade 2	Grade 3	Grade 4	Grade 5
Diarrhea	7	2	5	0	0	0
Colitis	12	2	8	1	1	0
Gastritis	1	0	1	0	0	0
Hepatitis	6	2	3	1	0	0
Skin rash	20	16	4	0	0	0
Pneumonitis	8	1	6	1	0	0
Thyroid dysfunction	18	4	14	0	0	0
Arthritis	3	2	1	0	0	0
Hypophysitis	1	1	0	0	0	0
Others	4	4	0	0	0	0
Total	80	34	42	3	1	0

**FIGURE 2:**
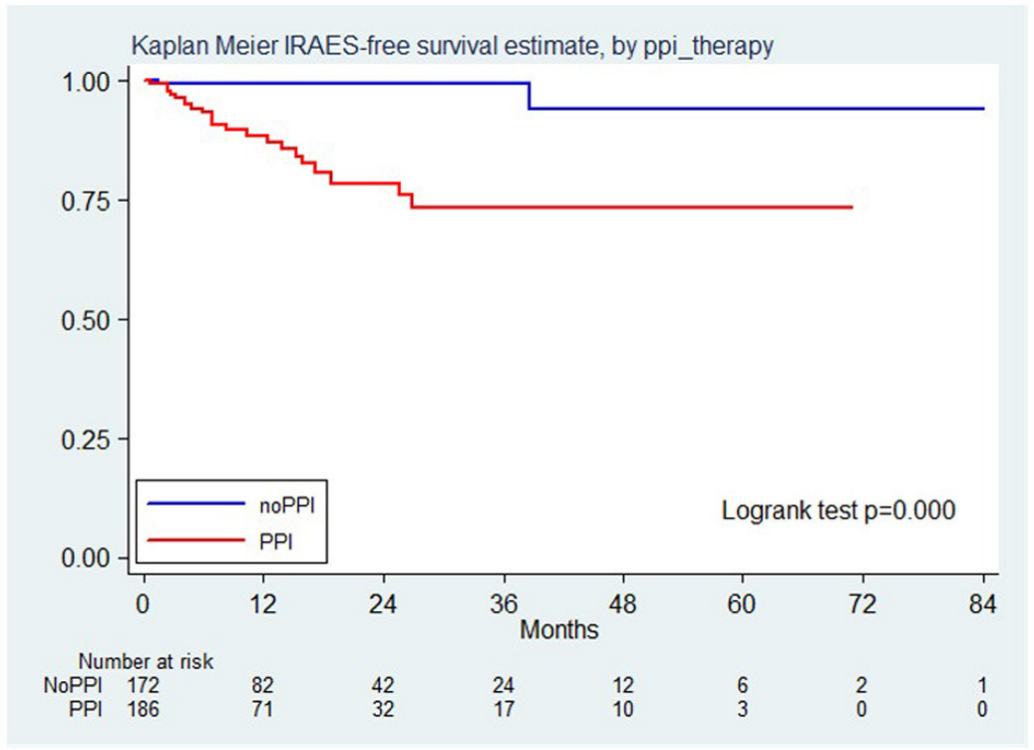
Kaplan Meier IRAES free survival estimate, by PPI therapy.

The incidence of irAEs was 5.4 events per 100 person year (95% CI 3.7–8.0). Twenty‐three exposed patients (92%) developed GI irAEs while only two unexposed patients (8%) developed GI irAEs ([HR] 13.22, 95% [CI] 3.11–56.10, *p* < 0.001). In particular, one patient developed G1 diarrhea and one patient G1 colitis. This HR was confirmed after weighting for the propensity score (HR 15.13 95% CI 3.22–71.03, *p* < 0.001; Figure 2).

### 3.4 Secondary endpoint: OS and exposure to PPIs

**FIGURE 3:**
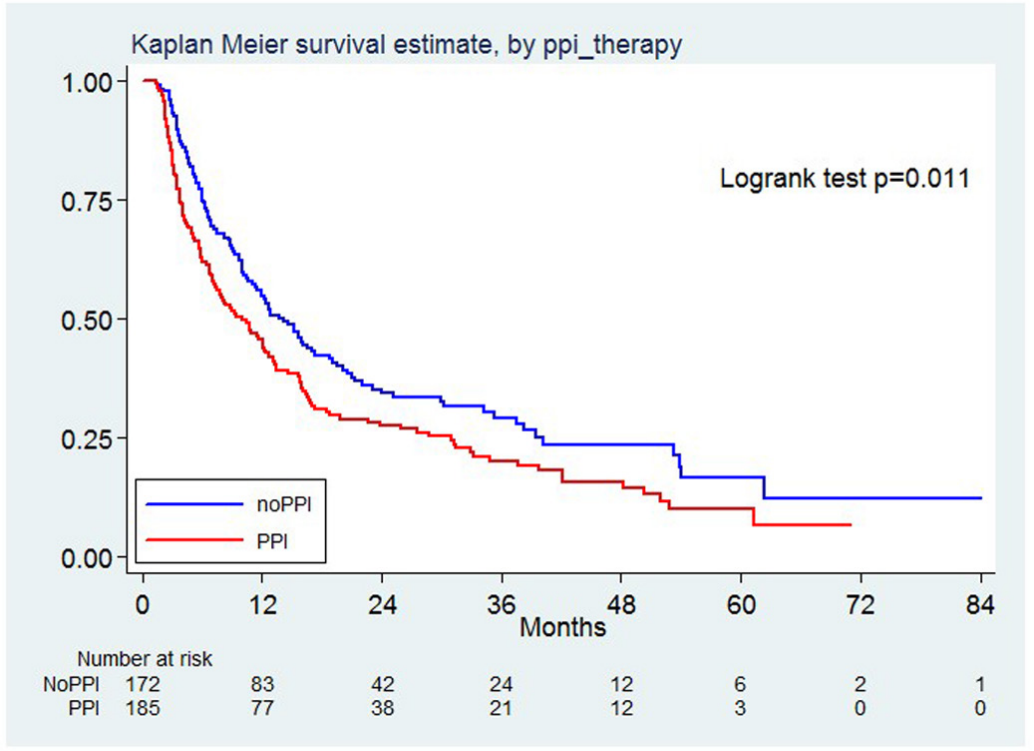
Kaplan Meier survival estimate, by PPI therapy.

Two‐hundred and sixty‐two patients died over a median follow‐up of 38 months (IQR 23–54). OS was 61.8% (95% CI 54.3%–68.4%) at 6 months and 45.6% (95% CI 38.2%–52.8%) at 12 months in PPI exposed patients. OS was 76.1% (95% CI 68.9%–81.7%) at 6 months and 55.3% (95% CI 47.4%–62.5%) at 12 months in PPI unexposed patients. Patients not treated with PPI had better OS than patients treated with PPI (HR 1.37, 95% CI 1.07–1.75, *p* = 0.011, Figure 3). After weighting, the propensity score showed that the use of PPI was a borderline nonsignificant risk factors for death [HR 1.25, 95% CI 0.99–1.59, *p* = 0.063].

### 3.5 Secondary endpoint: PFS and exposure to PPIs

**FIGURE 4:**
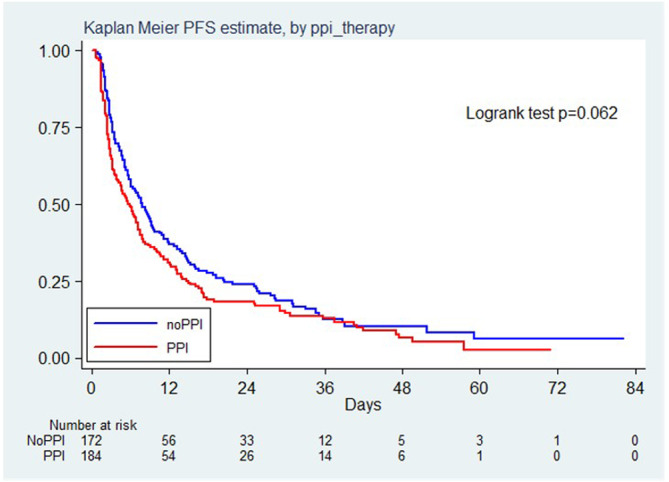
Kaplan Meier PFS estimate, by PPI therapy.

Disease progression were observed in 305 patients, corresponding to a rate of 87 per 100 person year. PFS were 48.9%, (95% CI 41.5%–55.9%) and 30.8% (95% CI 24.3%–37.7%) at 6 and 12 months, respectively, in PPI exposed patients. PFS were 57.4%, (95% CI 49.7%–64.4%) and 36.8% (95% CI 29.5%–44.1%) at 6 and 12 months, respectively, in PPI unexposed patients. There was no significant difference according to PPI use about PFS (HR 1.24, 95% CI 0.99–1.55, *p* = 0.064 (Figure 4). After weighting, the propensity score showed that the use of PPI was not a significant risk factor for disease progression (HR 1.03, 95% CI 0.83–1.28, *p* = 0.78).

## 4 DISCUSSION

This is one of the first studies assessing the impact of PPIs on the onset of GI irAES in patients with solid tumors undergoing ICIs. PPIs have a well‐known dysbiotic effect, as demonstrated in previous reports.^13^, ^14^ Due to these alterations in the composition of the GM, PPIs might be able to modify the response to ICIs.^15^ In contrast, other authors have not demonstrated an impact of PPIs on the response to ICIs.^16^

Since PPIs can modulate GM and GI irAEs depending on altered bio‐diversity of GM we hypothesized that patients exposed to PPIs had a higher risk of developing GI irAEs. During the period of the collection of data 25 GI irAES were reported. The majority of our patients (92%) were PPI exposed (HR 13.22, 95% CI 3.11–56.10, *p* < 0.0001). Importantly, this HR is confirmed after weighting for the propensity score (HR 15.13 95% CI 3.22–71.03, *p* < 0.0001). Our results are in line with those from a recent study that demonstrated the PPI use as a risk factor for chronic immune‐mediated diarrhea and colitis.^17^ PPIs can modify the composition of GM by, altering pH and modulating the immune response through their effect to on neutrophils, cytokines and natural killer cells (NK cells).^18^ The compositional and functional alterations in GM lead to intestinal barrier breakage with an increased translocation of toxins and inflammatory factors that may be able to alter dynamically the immunological profile in a pro‐inflammatory direction. These cytokines, including TNFα and IL‐10, may shift the threshold of immune subsets activation within the tumor micro‐environment, thereby resulting in augmented adaptive immune responses.^19^ The exact mechanism of PPI exposure and increased risk of GI irAEs remains unknown, but if these data will be confirmed by larger prospective cohorts with GM analysis, the clinicians should use PPIs more carefully and only if strictly indicated. In fact, there is a high percentage of inappropriate PPI prescription. More than half of PPIs prescribed among our patients were not clearly motivated. This evidence is in line with other papers.^20^, ^21^

In our cohort, concomitant PPI use seems to have a detrimental impact on PFS and OS, even if statistical significance is not reached. Two recent meta‐analyses suggested that PPIs are significantly associated with poorer OS and PFS for cancer patients treated with ICIs.^22^, ^23^ The dysbiosis caused by PPIs may modulate the antitumor immunity and inflammation, as reported by other authors.^24^ Indeed, other comorbidities and polypharmacy, increasing the risk of drug–drug interactions, may constitute additional detrimental factors in PPI‐exposed individuals.

Some limitations of the study must be mentioned. First, this is a retrospective single‐center study that might have been affected by reporting bias and some missing data regarding baseline and previous co‐medications. Second, we did not perform a GM analyses, as this will be part of a larger, prospective study that will soon start. Lastly, our results should be carefully interpreted in the light of the enrolling center characteristics (i.e., tertiary referral center), the relatively small sample size, and the tumor heterogeneity. We recognize that the number of patients in the final analysis is really too small to draw significant conclusions, nonetheless, despite these limitations, our data seem to suggest that chronic PPI use may be associated with an increased risk of GI irAES.

## 5 CONCLUSIONS

In conclusion, in this study we have shown that PPI use is associated with an increased risk of GI irAEs. Prospective studies are needed to confirm these findings.In all cases, we recommend a careful use of PPIs in this setting, only in case a clear indication is evident.

## AUTHOR CONTRIBUTIONS

**Angioletta Lasagna:** Conceptualization (equal); data curation (equal); investigation (equal); validation (equal); visualization (lead); writing – original draft (lead); writing – review and editing (lead). **Federica Mascaro:** Data curation (equal). **Simone Figini:** Data curation (equal). **Sara Basile:** Visualization (equal). **Giulia Gambini:** Methodology (equal); software (equal). **Catherine Klersy:** Formal analysis (lead); methodology (equal); software (equal). **Marco Vincenzo Lenti:** Conceptualization (equal); investigation (equal). **Antonio Di Sabatino:** Supervision (equal). **Alice Di Benedetto:** Investigation (equal). **Monica Calvi:** Supervision (equal). **Raffaele Bruno:** Supervision (equal). **Paolo Sacchi:** Investigation (equal); supervision (equal). **Paolo Pedrazzoli:** Funding acquisition (lead); project administration (equal); resources (lead).

## FUNDING INFORMATION

This work was partially supported by Ricerca Corrente grant no 41087/2017, Fondazione IRCCS Policlinico San Matteo.

## CONFLICT OF INTEREST STATEMENT

None to declare.

## ETHICS STATEMENT

This Study was approved by the local Ethics Committee (Comitato Etico Area Pavia) and Institutional Review Board (P‐0004914/23) according to the ethical guidelines of the 1975 Declaration of Helsinki.

## CONSENT

All the subjects signed, before the initiation of treatment, an informed consent provided by the Fondazione IRCCS Policlinico San Matteo at hospitalization.

## Data Availability

The data generated i this study are available upon request from the corresponding author.
